# Sertaconazole provokes proapoptotic autophagy via stabilizing TRADD in nonsmall cell lung cancer cells

**DOI:** 10.1002/mco2.102

**Published:** 2021-12-16

**Authors:** Wenhui Zhang, Li Zhou, Siyuan Qin, Jingwen Jiang, Zhao Huang, Zhe Zhang, Xiyu Zhang, Zheng Shi, Jie Lin

**Affiliations:** ^1^ Department of Medical Oncology The Second Affiliated Hospital of Kunming Medical University Kunming P.R. China; ^2^ State Key Laboratory of Biotherapy and Cancer Center West China Hospital, and West China School of Basic Medical Sciences & Forensic Medicine, Sichuan University and Collaborative Innovation Center for Biotherapy Chengdu P.R. China; ^3^ West China School of Basic Medical Sciences & Forensic Medicine Sichuan University Chengdu P.R. China; ^4^ Clinical Medical College & Affiliated hospital of Chengdu University Chengdu University Chengdu P.R. China

**Keywords:** apoptosis, autophagy, NSCLC, sertaconazole, TRADD

## Abstract

Nonsmall cell lung cancer (NSCLC) is one of the most commonly diagnosed and lethal cancers characterized by relatively low overall cure and poor survival rates with great challenge for consistent effective clinical treatment. Here we demonstrated that the antifungal sertaconazole displays potent anti‐NSCLC effect by promoting apoptosis in vitro and in vivo. Further studies found that sertaconazole induces complete autophagic flux, which contributes to sertaconazole‐induced apoptosis and subsequent growth suppression in NSCLC cells. Further studies demonstrated that sertaconazole provokes TNF receptor type 1 associated death domain protein (TRADD) expression via stabilizing it from ubiquitination‐mediated degradation, which results in Akt dephosphorylation and thereby triggers proapoptotic autophagy in NSCLC cells. Moreover, we found that TRADD suppression reverses sertaconazole‐induced proapoptotic autophagy and relieves growth suppression, indicating the vital role of TRADD‐regulated proapoptotic autophagy in the anti‐NSCLC activity of sertaconazole. In summary, our findings suggest that sertaconazole could be a highly promising anti‐NSCLC drug by triggering proapoptotic autophagy via stabilizing TRADD, which may provide a new potential therapeutic option for patients with NSCLC.

## Graphical abstract

Sertaconazole promotes TRADD expression via stabilizing it from ubiquitination‐mediated degradation, which induces Akt dephosphorylation and subsequent proapoptotic autophagy, thus suppressing NSCLC cell growth.

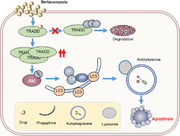

## 1 INTRODUCTION

Lung cancers rank the first in terms of incidence and contribute to nearly 30% of all cancer deaths in China, among which nonsmall cell lung cancer (NSCLC) accounts for about 85% of all lung cancers.^1^, ^2^, ^3^ Although smoking cessation and early diagnosis followed by surgical resection have greatly reduced the mortality rates of NSCLC, molecular targeted therapy, platinum‐based chemotherapy, or immunotherapy applied to patients with advanced NSCLC just displays compromised effects due to remarkable drug resistance mainly mediated by cancer driver gene alteration, epigenetic alteration, and tumor heterogeneity, which was further manifested by poor response to follow‐up therapy and unfavorable cancer relapse.^4^, ^5^, ^6^, ^7^ In this context, the survival rate of NSCLC patients is still relatively low and new molecular targets or therapeutic agents are urgently needed for developing novel treatment options and therefore achieving better clinical outcome.

Drug repurposing, which refers to identifying new indications of approved or investigational drugs, has attracted much attention for developing novel therapeutic options to treat several types of disease, especially in cancer.^8^, ^9^ Repositioning the large pool of noncancer drugs has become a novel approach for exploring more effective, cheaper, and safer drugs to treat cancers.^10^ Indeed, studies in the last decade have demonstrated that many kinds of non‐cancer drugs, including antibiotics, antihyperglycemic drugs, antifungal, and antiparasitic agents, displayed potent anticancer activity against several types of cancer.^11^ For example, epidemiological and experimental studies have identified metformin, a standard clinical drug used for type 2 diabetes treatment, as a potential antineoplastic drug against several cancers including gastrointestinal cancer, liver cancer, and breast cancer.^12^, ^13^, ^14^, ^15^ And furthermore, several clinical trials have been conducted to evaluate its potential application in cancer patients,^16^, ^17^, ^18^ implying drug repurposing as an effective strategy and possible future direction for drug discovery. Nevertheless, few repurposed drugs are included in cancer guidelines so far, and many of them are under investigation.^19^ Thus, more candidates should be explored by drug repurposing for further investigation.

Sertaconazole, an imidazole‐type antifungal agent, displays considerable antifungal activity against pathogenic fungi, including yeast‐like fungi, dermatophytes, and other filamentous fungi.^20^, ^21^ Just like other azoles, sertaconazole disrupts mycelial growth and replication by inhibiting ergosterol synthesis.^22^ Besides, sertaconazole also shows anti‐inflammatory and anti‐itch activity by activating the p38‐COX‐2‐PGE_2_ pathway in keratinocytes and human peripheral blood mononuclear cells.^23^, ^24^ Recent evidence has suggested that the imidazole‐based antifungal drugs could display cytotoxicity against diverse human cancers, for instance, miconazole has been found inducing apoptosis and suppressing the growth of bladder and colorectal cancer cells.^25^

Although the antifungal sertaconazole has recently been reported to inhibit HeLa cell growth,^26^ little is known about the anticancer effect of sertaconazole against NSCLC and the involved mechanisms are poorly investigated. In the present study, we sought to repurpose sertaconazole for the treatment of NSCLC and investigate the efficacy against NSCLC to explore an alternative therapeutic drug for NSCLC treatment.

## 2 RESULTS

### 2.1 Sertaconazole inhibits NSCLC cell growth in vitro and in vivo

**FIGURE 1:**
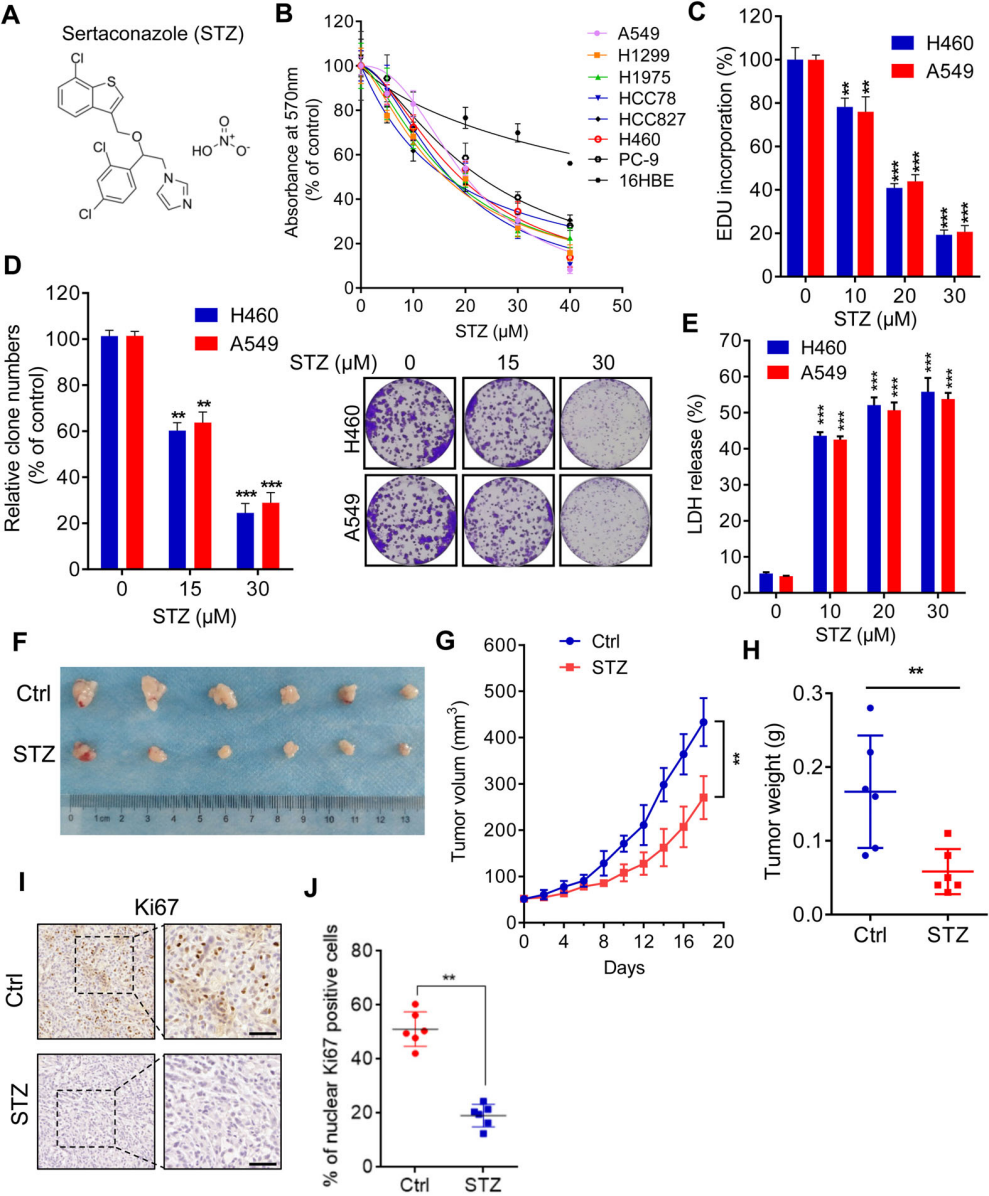
Sertaconazole inhibits NSCLC cell growth in vitro and in vivo. (A) The chemical structure of sertaconazole (STZ). (B) Cell growth of human NSCLC cell lines and the bronchial epithelial cell line 16HBE treated with sertaconazole at indicated concentrations. (C, D) A549 and H460 cells were treated with sertaconazole for 48 h. Cell proliferation was determined by EdU incorporation (C) and colony formation (D). (E) Cells were incubated with sertaconazole for 48 h, and cytotoxicity was detected by analyzing the release of LDH. (F–H) A549 cells were subcutaneously inoculated into nude mice. Mice were intraperitoneally injected with vehicle or sertaconazole. Image (F), volume (H), and weight (G) of tumor xenografts were measured. (I, J) Immunohistochemical staining of Ki67 in tumor xenografts. Scale bar, 50 μm. All experiments were repeated at least three times. Statistic method: 2‐tailed Student's t‐test. Data are means with SD. **p < 0.01, ***p < 0.001

To examine the antitumor activity of sertaconazole against NSCLC cells, cell growth was detected in several kinds of NSCLC cells after exposure of sertaconazole treatment. As depicted in Figure 1A and B, treatment of sertaconazole for 48 h obviously suppressed the growth of NSCLC cell lines (including A549, H1299, H1975, HCC78, HCC827, H460, and PC‐9). In contrast, the bronchial epithelial cell line 16HBE showed higher tolerance to sertaconazole treatment with nearly threefold IC50 value than that of cancer cells (Figure S1A). The proliferation of NSCLC cells was also obviously decreased upon sertaconazole treatment, supported by results from EdU assay (Figure 1C) and colony formation assay (Figure 1D). Moreover, we performed LDH release assay to monitor the cytotoxicity of sertaconazole. As expected, sertaconazole treatment markedly promoted the release of LDH in A549 and H460 cells, indicating an obvious cytotoxicity induced by sertaconazole (Figure 1E). Collectively, these data indicate that sertaconazole suppresses NSCLC cell growth in vitro.

To evaluate the antitumor effect of sertaconazole against NSCLC in vivo, a tumor xenograft model of NSCLC was established by subcutaneously inoculating A549 cells into the BALB/c nude mice. As depicted in Figure 1F–H, the growth rate, size and weight of tumors were significantly inhibited in sertaconazole group compared with the vehicle group. In agreement with these observations, sertaconazole treatment also resulted in relatively weaker staining of Ki67, a recognized indicator of proliferation, compared with the vehicle group via immunohistochemical (IHC) staining (Figure 1I and J). In addition, no significant pathologic features change of major organs and weight loss were observed in response to sertaconazole treatment, indicating that sertaconazole has no evident toxicity in mice (Figure S1B and C). Collectively, these results demonstrate that sertaconazole suppresses NSCLC cell growth in vitro and in vivo.

### 2.2 Sertaconazole suppresses NSCLC cell growth by triggering apoptosis

**FIGURE 2:**
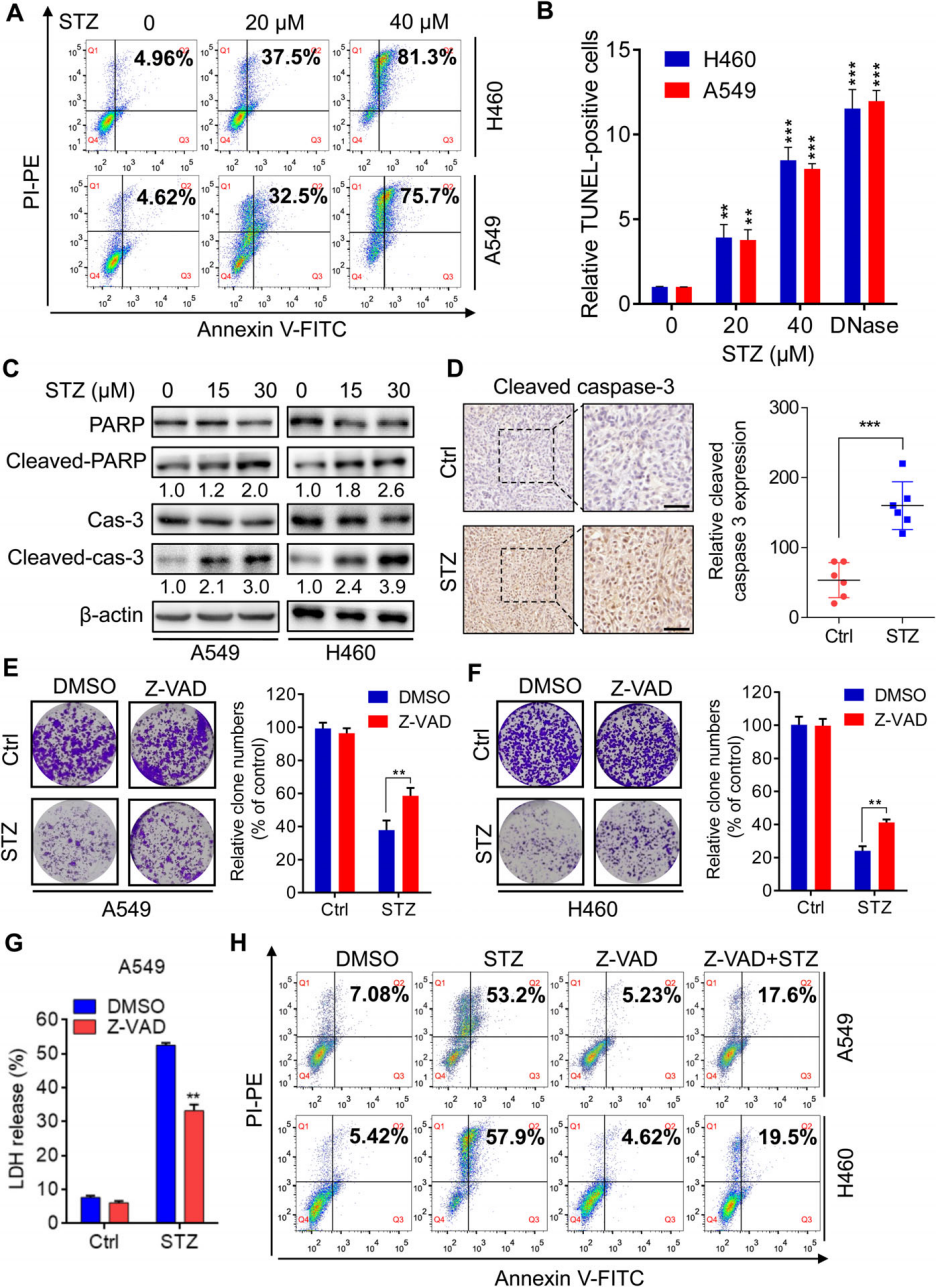
Sertaconazole suppresses NSCLC cell growth by triggering apoptosis. (A) NSCLC cells were treated with sertaconazole for 48 h, and flow cytometry was conducted to detect the apoptotic cells. (B) NSCLC cells were treated as in (A), and TUNEL assay was performed to detect the apoptotic cells. Statistic method: 2‐tailed Student's *t*‐test. (C) Immunoblot analysis of cleaved caspase 3, caspase 3, PARP, and cleaved PARP level in NSCLC cells treated with sertaconazole for 24 h. (D) Immunohistochemical staining of cleaved caspase 3 in tumor xenografts. Scale bar, 50 μm. Statistic method: 2‐tailed Student's *t*‐test. (E, F) Colony formation of A549 and H460 cells treated with sertaconazole and in combination with or without Z‐VAD for 2 weeks. Statistic method: Two‐way ANOVA. G) Analysis of LDH release of A549 cells treated with sertaconazole in combination with or without Z‐VAD. Statistic method: Two‐way ANOVA. (H) A549 and H460 cells were incubated with sertaconazole in combination with or without Z‐VAD for 48 h. Flow cytometry was conducted to detect the apoptotic cells. All experiments were repeated at least three times. Data are means with SD. **p < 0.01, ***p < 0.001

Then, we investigated the mechanism by which sertaconazole suppresses NSCLC cell growth. The inhibitors of different forms of cell death were used to evaluate their effects on NSCLC cell growth upon sertaconazole treatment. As shown in Figure S2A, combination of Z‐VAD (one of the apoptosis inhibitors) or chloroquine (CQ, an inhibitor of autophagy) with sertaconazole relieved the inhibitory effect of sertaconazole on NSCLC cell growth, while other inhibitors, including ferrostatin‐1 (Fer‐1, one of the ferroptosis inhibitors) and necrostatin‐1 (Nec, one of the necroptosis inhibitors), displayed no significant effect on NSCLC cell growth after treatment of sertaconazole. As apoptosis is a well‐known cell death caused by chemotherapeutic agents,^27^ based on such findings, we first tested whether sertaconazole induces apoptosis in NSCLC cells. Flow cytometric analysis of Annexin V/PI staining was conducted to detect apoptosis induction after sertaconazole treatment. As depicted in Figure 2A and Figure S2B, treatment with sertaconazole for 48 h showed significant induction of apoptosis in H460 and A549 cells. We also used TUNEL assays to evaluate the proapoptotic effect of sertaconazole in NSCLC cells (Figure 2B). The induction of apoptosis was further demonstrated by accumulated cleaved‐caspase 3 and cleaved‐PARP in A549 and H460 cells after sertaconazole treatment (Figure 2C). Moreover, significant apoptosis induction was found in tumors from sertaconazole group as evidenced by stronger cleaved‐caspase 3 intensity (Figure 2D). Concomitantly, treatment with apoptosis inhibitor Z‐VAD rescued sertaconazole‐induced suppression of NSCLC cell growth, supported by the results from colony formation assays (Figure 2E and F). Z‐VAD treatment could also counteract sertaconazole‐induced cytotoxicy and apoptosis, which was supported by the LDH release assay (Figure 2G and Figure S2C) and flow cytometry analysis (Figure 2H and Figure S2D). In summary, these findings demonstrate that sertaconazole induces apoptosis to retard NSCLC growth.

### 2.3 Sertaconazole promotes complete autophagic flux in NSCLC cells

**FIGURE 3:**
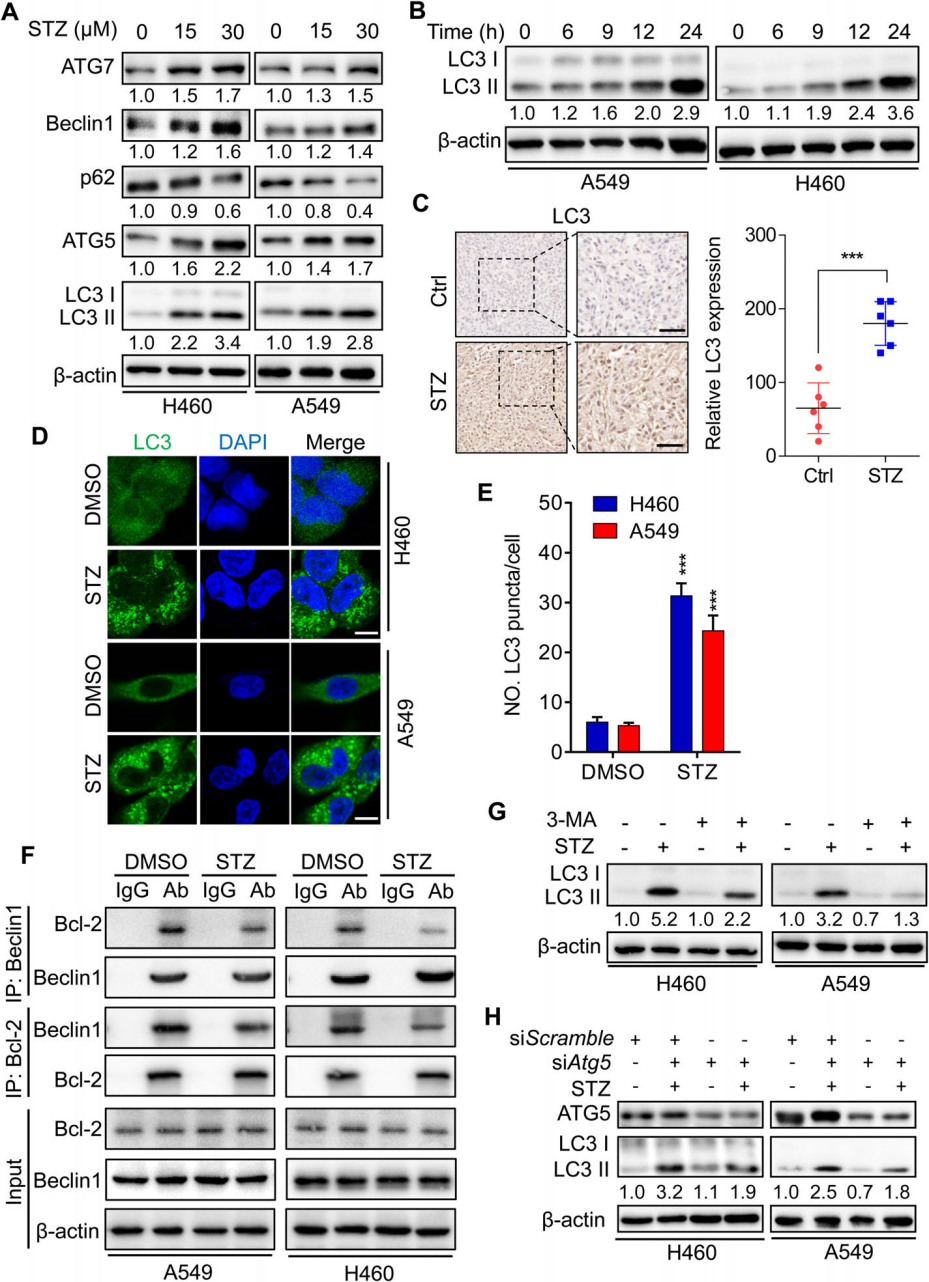
Sertaconazole induces autophagy in NSCLC cells. (A) Immunoblotting of ATG7, Beclin 1, p62, ATG5, and LC3 turnover in A549 and H460 cells treated with sertaconazole for 24 h. (B) Immunoblotting of LC3 turnover in A549 and H460 cells treated with the indicated times of sertaconazole. (C) Immunohistochemical staining of LC3 in tumor xenografts. Scale bar, 50 μm. Statistic method: 2‐tailed Student's *t*‐test. (D, E) Immunofluorescent analysis of endogenous LC3 puncta in cells treated with or without sertaconazole for 24 h. Scale bar, 10 μm. Statistic method: 2‐tailed Student's *t*‐test. (F) The interaction between Beclin 1 and Bcl‐2 in response to sertaconazole treatment was analyzed by reciprocal coimmunoprecipitation assay. (G) A549 and H460 cells were treated with sertaconazole in the absence or presence of 3‐MA for 24 h. The LC3 turnover was detected by immunoblotting. (H) Cells were transfected with si*Atg5* or si*Scramble*, followed by treatment with sertaconazole for 24 h. The LC3 turnover was detected by immunoblotting. All experiments were repeated at least three times. Data are means with SD. ***p < 0.001

As was conveyed by Figure S2A, we surprisingly found that combination of autophagy inhibitor CQ with sertaconazole also relieved the growth inhibition of NSCLC cells induced by sertaconazole, implying the potential role of autophagy in sertaconazole‐induced growth suppression. Considering that autophagy has been found to play key roles in drug‐induced cancer cell death,^28^, ^29^, ^30^, ^31^, ^32^ thus we are interest to investigate whether sertaconazole modulates autophagy to promote apoptosis in NSCLC cells. To confirm this hypothesis, we first detected the expression of autophagy proteins in NSCLC cells after treatment of sertaconazole. As depicted in Figure 3A and B, sertaconazole treatment showed obvious induction of autophagy as evidenced by elevated levels of lipidated LC3‐II, the classical markers of autophagy in A549 and H460 cells. This autophagy induction by sertaconazole was further confirmed by increased endogenous LC3 puncta in sertaconazole‐treated cells and increased LC3 intensity in tumors from sertaconazole group (Figure 3C–E). As the accumulation of LC3‐II may result from either enhanced autophagy initiation or impaired degradation in late stage of autophagy, we next explored the underlying mechanisms of sertaconazole‐induced autophagosome accumulation in NSCLC cells. As presented in Figure 3A, the expression of autophagic machinery components (ATG7, Beclin1 and ATG5) increased in sertaconazole‐treated A549 and H460 cells, indicating the initiation of autophagy. This was further validated by the finding that sertaconazole treatment resulted in increased dissociation of Beclin1 with Bcl‐2 (Figure 3F), which is an essential event during autophagy initiation.^33^, ^34^ In accordance with these observations, pharmacological inhibition of autophagy using the class III PI3K inhibitor 3‐MA^35^ or si*Atg5* silencing, significantly reduced the elevation of LC3‐II levels and the accumulation of LC3 puncta in sertaconazole‐treated A549 and H460 cells (Figure 3G and H and Figure S3A–H). Overall, these results indicate that sertaconazole promotes the initiation of autophagy in NSCLC cells. Interestingly, sertaconazole treatment displayed no obvious autophagy induction in the bronchial epithelial cell line 16HBE, implying that sertaconazole‐induced autophagy might be tumor‐specific (Figure S3I and J).

**FIGURE 4:**
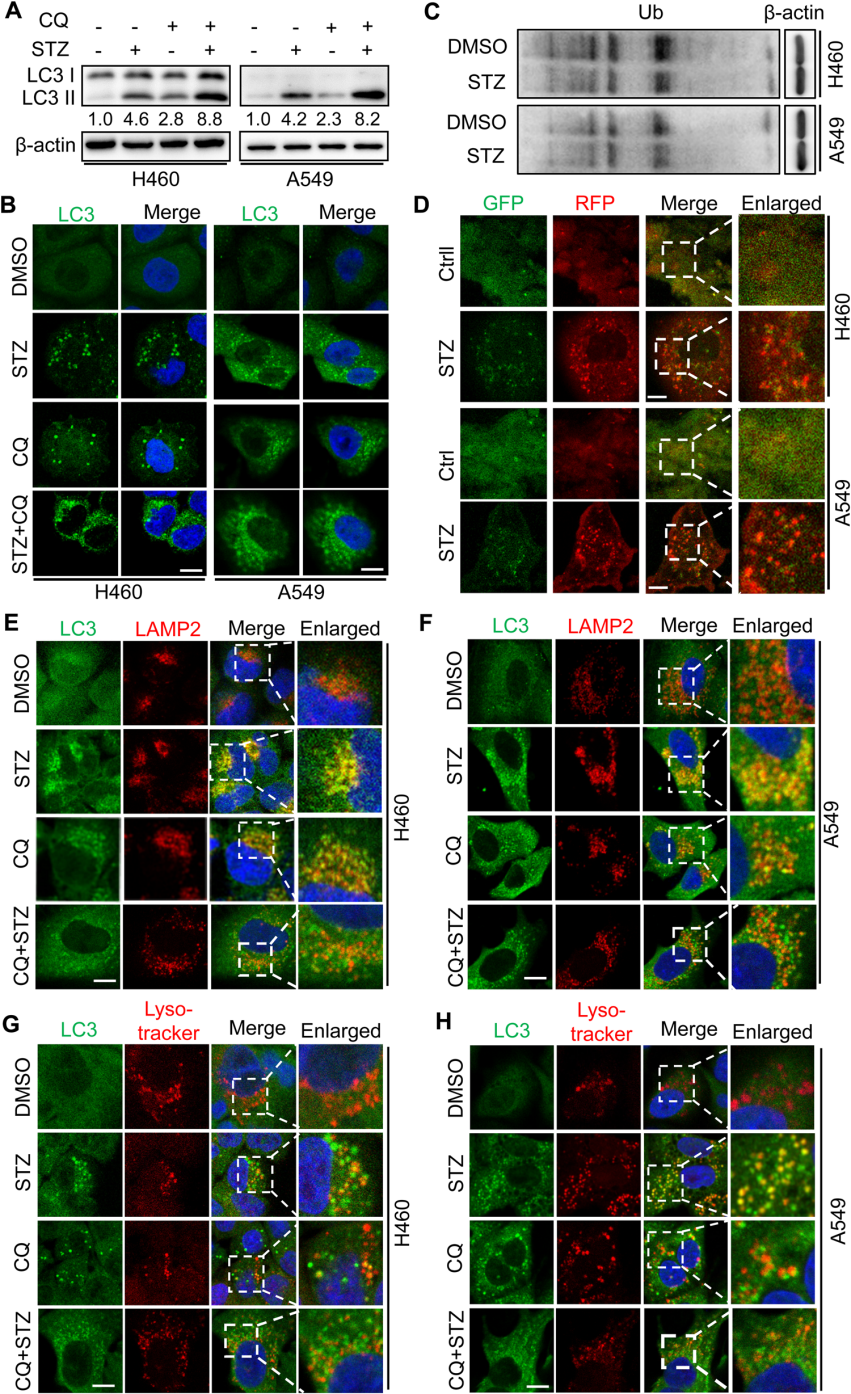
Sertaconazole promotes autophagy flux in NSCLC cells. (A) A549 and H460 cells were incubated with sertaconazole in the absence or presence of CQ for 24 h. The LC3 turnover was detected by immunoblotting. (B) A549 and H460 cells were treated as in (A). Endogenous LC3 puncta were detected by immunofluorescent analysis. Scale bar, 10 μm. (C) Immunoblotting of Ub in A549 and H460 cells treated with or without sertaconazole. (D) Immunofluorescence of NSCLC cells transfected with RFP‐GFP‐LC3 and treated with or without sertaconazole. Scale bar, 10 μm. (E, F) The colocalization of LC3 with LAMP2 after the treatment of sertaconazole for 24 h. Scale bar, 10 μm. (G, H) The colocalization of LC3 with lysosome was quantitated by Lysotracker staining after the treatment of sertaconazole for 24 h. Scale bar, 10 μm. All experiments were repeated at least three times

We next determined to explore whether sertaconazole induces complete autophagic flux. As shown in Figure 3A, the level of p62 which may be downregulated due to autolysosome degradation, was decreased after sertaconazole treatment. In addition, combination treatment of sertaconazole with CQ further increased LC3‐II levels and LC3 puncta (Figure 4A and B and Figure S4A). Consistently, ubiquitinated protein conjugates were reduced after treatment with sertaconazole (Figure 4C). Moreover, using a tandem mRFP‐GFP‐LC3 constructs, there emerged increased autolysosomes (red dots, RFP^+^ GFP^−^) accompanied by reduced autophagosomes (yellow dots, RFP^+^ GFP^+^) in STZ‐treated A549 and H460 cells (Figure 4D and Figure S4B). We also determined the colocalization of autophagosome (LC3) with lysosome (LAMP2) in sertaconazole‐treated NSCLC cells. As depicted in Figure 4E and F and Figure S4C, sertaconazole treatment led to marked colocalization of LC3 with LAMP2, indicating the formation of autolysosome. To further verify the fusion of autophagosome with lysosome in NSCLC cells treated with sertaconazole, we detected the colocalization of lysosome with LC3 by LysoTracker staining. These results suggested that treatment with sertaconazole markedly promoted colocalization of lysosome with LC3, reconfirming the fusion of autophagosome with lysosome (Figure 4G and H and Figure S4D). Moreover, in response to STZ treatment, the lysosomal acidity was monitored by acridine orange (AO) staining (Figure S4E). In summary, these data suggest that sertaconazole promotes complete autophagic flux in NSCLC cells.

### 2.4 Sertaconazole‐induced autophagy enhances its proapoptotic effect to suppress NSCLC cell growth

**FIGURE 5:**
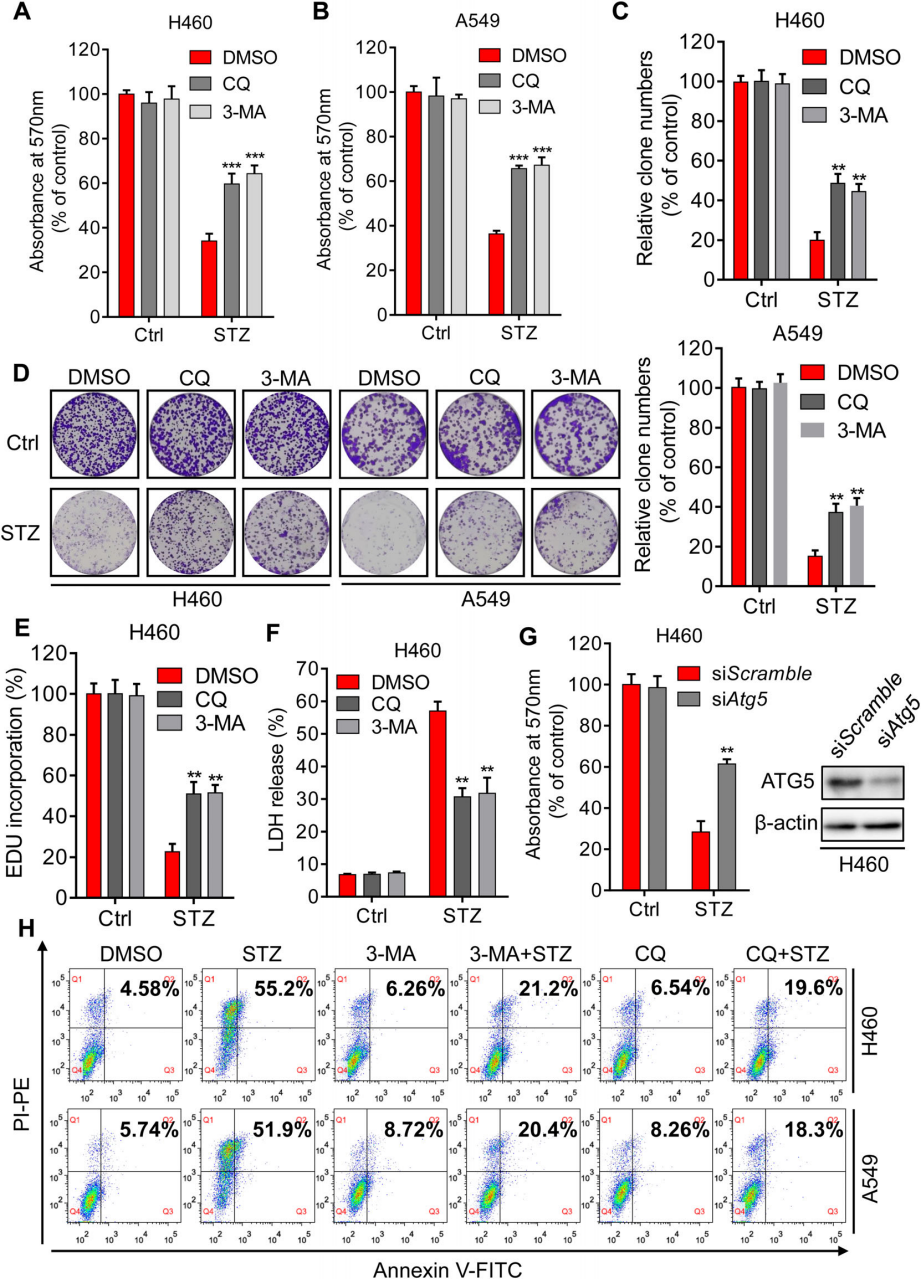
Sertaconazole‐induced autophagy enhances its proapoptotic effect to suppress NSCLC cell growth. (A, B) MTT assay of A549 and H460 cells treated with CQ or 3‐MA in the presence or absence of sertaconazole for 48 h. Statistic method: Two‐way ANOVA. (C, D) Colony formation of NSCLC cells treated with sertaconazole in combination with or without 3‐MA or CQ. Statistic method: Two‐way ANOVA. (E) EdU incorporation assay was performed to detect the proliferation of H460 cells treated with sertaconazole in combination with or without 3‐MA or CQ. Statistic method: Two‐way ANOVA. (F) H460 Cells were subjected to sertaconazole in combination with or without 3‐MA or CQ, and cytotoxicity was detected by analyzing the release of LDH. Statistic method: Two‐way ANOVA. (G) H460 cells were transfected with si*Atg5* or si*Scramble*, followed by treatment with or without sertaconazole for 48 h. Cell growth was detected by MTT assay. Statistic method: Two‐way ANOVA. H) A549 and H460 cells were treated with sertaconazole in the absence or presence of CQ or 3‐MA for 48 h, and flow cytometry was performed to evaluate the apoptotic cells. All experiments were repeated at least three times. Data are means with SD. **p < 0.01, ***p < 0.001

As was illustrated in Figure S3I, sertaconazole treatment had no obvious effect on autophagy in the bronchial epithelial cell line 16HBE; we thus asked whether this tumor‐specific autophagy was required for the apoptosis induction and anti‐NSCLC effect of sertaconazole. To test this hypothesis, NSCLC cells were incubated with sertaconazole in combination with autophagy inhibitor 3‐MA or CQ. The MTT assays revealed that combinational use of autophagy inhibitors with sertaconazole partially restored sertaconazole‐induced growth inhibition in NSCLC cells (Figure 5A and B). Consistently, the cell proliferation of A549 and H460 was also rescued upon combinational treatment, which was supported by the colony formation assay (Figure 5C and D) and EdU incorporation assay (Figure 5E and Figure S5A). Moreover, combinational use of 3‐MA or CQ together with sertaconazole also reduced the cytotoxicity of sertaconazole in NSCLC cells by analysis of LDH release (Figure 5F and Figure S5B). Besides pharmacological inhibition of autophagy, we also performed siRNA‐mediated silencing of autophagy genes *Atg5* and *BECN1* to further strengthen this conclusion. In line with the above findings, autophagy inhibition by *Atg5* or *BECN1* silencing partially offset the growth suppression of NSCLC cells induced by sertaconazole (Figure 5G and Figure S5C–E). Of note, 3‐MA or CQ treatment also relieved sertaconazole‐induced apoptosis in NSCLC cells (Figure 5H and Figure S5F), implying the induction of proapoptotic autophagy after sertaconazole treatment. Collectively, these results demonstrate that sertaconazole‐induced proapoptotic autophagy contributes to its anti‐NSCLC effect.

### 2.5 Sertaconazole induces autophagy by stabilizing TRADD in NSCLC cells

Next, we intend to explore the mechanism underlying sertaconazole‐induced autophagy. Previously studies have demonstrated that Akt/mTOR signaling functions as one of the classical negative modulators of apoptosis and autophagy triggered by anticancer drugs,^36^, ^37^ we thus detect whether sertaconazole inhibits the Akt/mTOR signaling in A549 and H460 cells. As found in Figure S6A and B, Akt/mTOR signaling was significantly restrained after sertaconazole treatment, as detected by decreased phosphorylation of Akt, mTOR, p70S6K, and 4EBP1 along with decreased phosphorylated Akt staining in tumors in sertaconazole group. To further ascertain whether Akt/mTOR signaling is associated with autophagy induced by sertaconazole, we transfected NSCLC cells with CA‐Akt (a constitutively active Akt) plasmids and found that sertaconazole‐induced LC3‐II levels and LC3 puncta were decreased after Akt reactivation in NSCLC cells (Figure S6C–E). These data suggest that Akt/mTOR signaling participates in sertaconazole‐induced autophagy in NSCLC cells.

**FIGURE 6:**
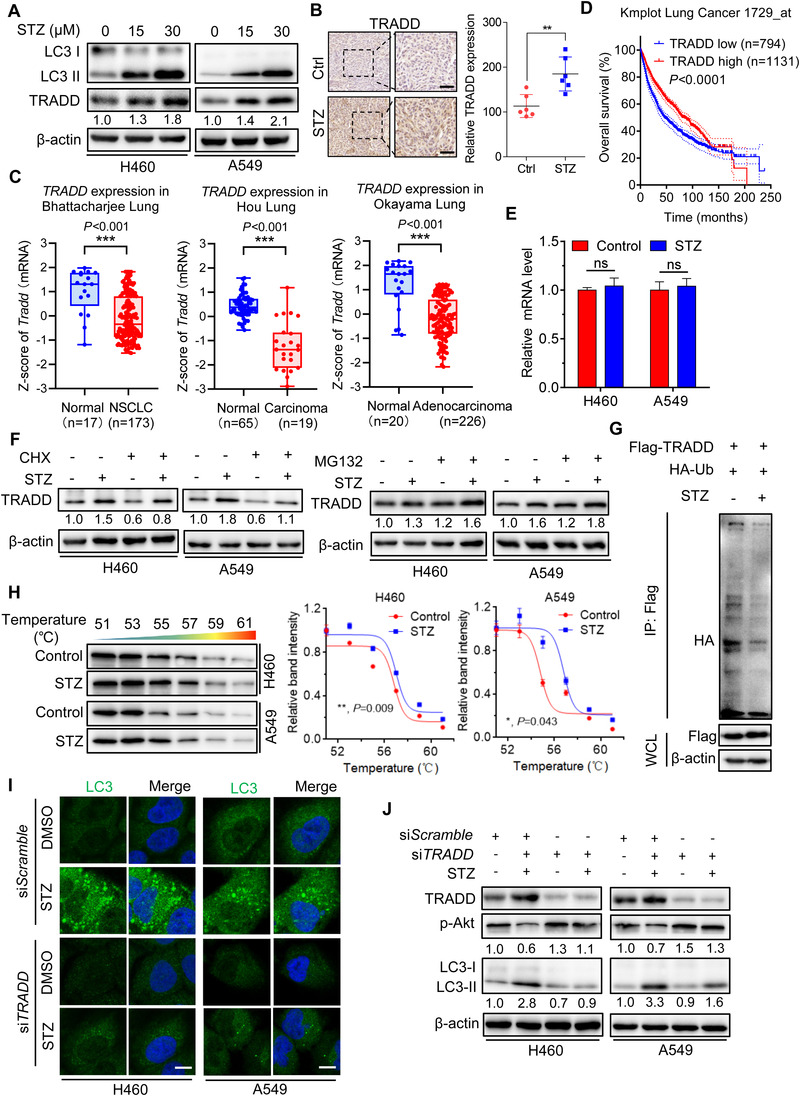
Sertaconazole induces autophagy by stabilizing TRADD in NSCLC cells. (A) Immunoblotting of TRADD expression in A549 and H460 cells treated with sertaconazole for 24 h. (B) IHC staining of TRADD in tumor xenografts. Scale bar, 50 μm. (C) Data represent the Z‐score of TRADD from Oncomine database. (D) Kaplan–Meier analysis of NSCLC patients from Kmplot database. (E) The relative mRNA levels of TRADD in cells treated with or without sertaconazole for 24 h was determined by qPCR analysis. (F) Immunoblotting analysis of TRADD protein level in cells treated with CHX or MG‐132 in the presence or absence of sertaconazole for 24 h. (G) 293T cells were transfected with Flag‐TRADD and HA‐tagged ubiquitin plasmids and then treated with sertaconazole for 24 h. Immunoprecipitation was used to detect the ubiquitination of TRADD. (H) Cellular thermal shift assay showing target engagement of TRADD by sertaconazole in A549 and H460 cells. (I, J) H460 and A549 cells were transfected with si*Scramble* or si*TRADD*, followed by treatment with sertaconazole for 24 h. Immunofluorescent analysis of endogenous LC3 puncta (I) and immunoblot analysis of LC3 turnover (J) and were performed. Scale bar, 10 μm. All experiments were repeated at least three times. Statistic method: 2‐tailed Student's t‐test. Data are means with SD. ns, not significant, *p < 0.05, **p < 0.01, ***p < 0.001

We next question how the AKT/mTOR signaling is regulated during sertaconazole treatment. Previous studies have demonstrated that Akt inactivation induced by antitumor drugs accompanies with the activation of death receptor signaling, mainly reflected the increased expression of Fas and TRADD (TNF receptor type 1 associated death domain protein).^38^, ^39^, ^40^, ^41^, ^42^ Interestingly, TRADD has recently been identified as an important regulator of both autophagy and apoptosis.^43^ Parallelly, we found sertaconazole regulated both apoptosis and autophagy process, implying the potential role of TRADD in response to sertaconazole treatment in NSCLC cells. To test this hypothesis, we detected the levels of TRADD protein in sertaconazole‐treated A549 and H460 cells and tumors from xenograft. As found in Figure 6A and B, the protein levels of TRADD were markedly increased after sertaconazole treatment both in vitro and in vivo. Consistent with previous study supporting the tumor suppressor role of TRADD,^44^ it was found that TRADD was downregulated in lung cancers by employing Oncomine database (Figure 6C). To further confirm the prognostic value of TRADD in NSCLC, we analyzed the correlation between TRADD expression and patient survival in Kmplot database and found high level of TRADD was correlated with improved patient survival (Figure 6D). Thus, these observations demonstrate that sertaconazole treatment upregulates TRADD expression in NSCLC cells and moreover, TRADD may serve as an indicator of improved prognosis.

Then, we intend to explore the mechanisms of TRADD upregulation under sertaconazole treatment. Interestingly, the mRNA level of TRADD was not significantly changed in sertaconazole‐treated A549 and H460 cells (Figure 6E). We thus questioned whether sertaconazole increases TRADD expression by reducing proteasomal degradation rather than promoting transcription. As expected, treatment of cycloheximide (CHX, a protein synthesis inhibitor) resulted in the inhibition of the rate of TRADD degradation in response to sertaconazole, while MG132 (a proteasome inhibitor) could further enhance sertaconazole‐induced TRADD expression (Figure 6F). This concept was further supported by data indicating an obviously reduced ubiquitin‐conjugated level of TRADD after sertaconazole treatment (Figure 6G). In addition, the cellular thermal shift assay found that TRADD was engaged and stabilized against thermal changes after sertaconazole treatment (Figure 6H). Together, our results suggest that sertaconazole protects TRADD from ubiquitin‐mediated degradation to upregulate its expression.

Next, we investigated whether TRADD was required for sertaconazole‐regulated autophagy in NSCLC cells using gain‐ and loss‐of‐function experiments. As presented in Figure 6I and J and Figure S7A–D, siRNA silencing of *TRADD* markedly reduced sertaconazole‐induced LC3B puncta accumulation and LC3B‐II turnover, as well as the colocalization of LC3 with lysosome in A549 and H460 cells. In contrast, exogenous overexpression of TRADD in NSCLC cells led to increased LC3B‐II level and endogenous LC3B puncta (Figure S7E–G), to a comparable level showed in NSCLC cells incubated with sertaconazole alone. Given the negative correlation between Akt and TRADD reported in the previous study, we thus investigated whether TRADD is required for sertaconazole‐induced inhibition of Akt phosphorylation in A549 and H460 cells. As shown in Figure 6J and Figure S7E and F, siRNA‐mediated *TRADD* silencing increased Akt phosphorylation, while exogenous overexpression of TRADD repressed Akt phosphorylation. Notably, the elevated levels of LC3 induced by TRADD overexpression could be counteracted by transfection of CA‐Akt, as evidenced by reduced LC3‐II turnover and LC3 puncta (Figure S7F and G). In summary, these data reveal that sertaconazole promotes autophagy by stabilizing TRADD in NSCLC cells.

### 2.6 TRADD is required for sertaconazole‐induced growth suppression in NSCLC cells

**FIGURE 7:**
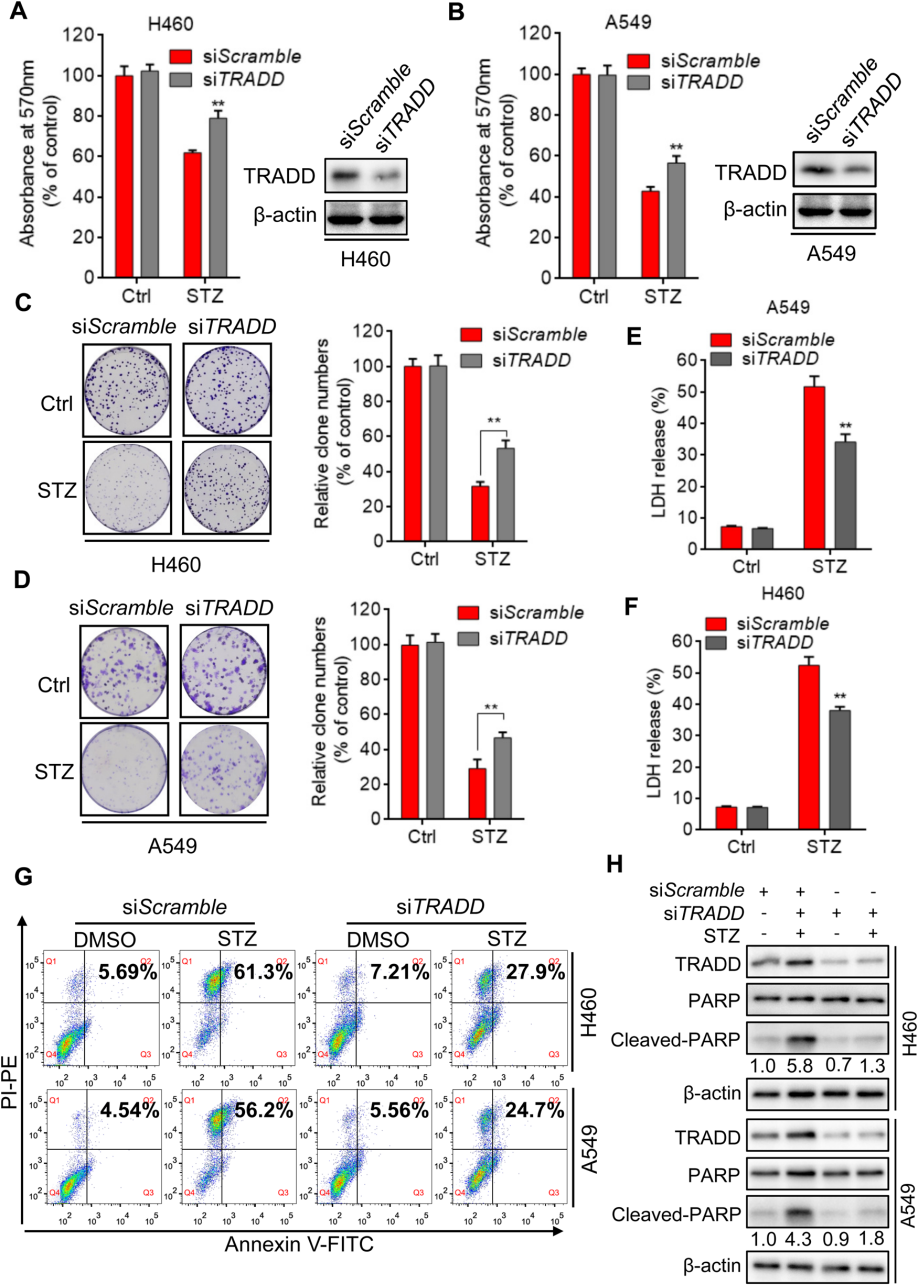
TRADD is required for sertaconazole‐induced NSCLC suppression. (A, B) H460 and A549 cells were transfected with si*Scramble* or si*TRADD*, followed by treatment with sertaconazole for 24 h. Cell growth was detected using MTT assay. (C, D) H460 and A549 cells were treated as in (A) and (B). Cell proliferation was monitored by colony formation assay. (E, F) H460 and A549 cells were treated as in (A) and (B). LDH release assay was used to detect the cytotoxic effect of sertaconazole. (G) H460 and A549 cells were transfected with si*Scramble* or si*TRADD*, followed by treatment with sertaconazole for 48 h. Flow cytometry was used to detect the apoptotic cells. (H) Immunoblotting of cleaved‐PARP level in cells transfected with si*TRADD* or si*Scramble* in the presence or absence of sertaconazole. All experiments were repeated at least three times. Data are means with SD. **p < 0.01

To further reveal the role of TRADD in the growth suppression induced by sertaconazole in NSCLC cells, A549 and H460 cells were transfected with si*Scramble* or si*TRADD*, followed by sertaconazole treatment for 24 h. The results from MTT assay found that silencing of *TRADD* rescued sertaconazole‐induced growth inhibition of NSCLC cells when compared with the si*Scramble* group (Figure 7A and B), indicating the vital role of TRADD in the anti‐NSCLC effect of sertaconazole. In agreement with this, TRADD suppression relieved sertaconazole‐induced inhibition on NSCLC cell proliferation, as evidenced by the colony formation assays (Figure 7C and D). In addition, the levels of released LDH were decreased after *TRADD* silencing compared with the si*Scramble* group (Figure 7E and F), suggesting attenuated cytotoxicity induced by sertaconazole. Moreover, flow cytometric analysis found that sertaconazole‐induced apoptosis was also attenuated after *TRADD* silencing in A549 and H460 cells (Figure 7G). Consistently, the level of cleaved‐PARP was decreased in cotreatment group, further indicating the essential role of TRADD in sertaconazole‐induced proapoptotic autophagy (Figure 7H). Collectively, these findings demonstrate that TRADD is essential for sertaconazole‐induced growth suppression of NSCLC cells.

## 3 DISCUSSION

Sertaconazole was approved by FDA as an antifungal agent.^45^ Recently, it has been found that sertaconazole induces toxicity in HeLa cells through inducing mitotic arrest and inhibiting microtubule assembly,^26^ but the underlying molecular mechanism requires further investigation. In the present study, we show that sertaconazole exerts obvious anticancer activity against NSCLC cells by triggering proapoptotic autophagy. Further investigations reveal that sertaconazole protects TRADD from ubiquitination‐mediated proteasomal degradation to increase its protein level, thereby decreasing the phosphorylation of Akt to induce autophagy in NSCLC cells. To our best knowledge, the findings of the present study demonstrate a previously unchartered effect of sertaconazole in suppressing NSCLC, which provides a newer perspective on NSCLC therapy with alternative therapeutic strategies of repurposing derisked “old” drugs.

Apoptosis (“self‐killing”) and autophagy (“self‐eating”) are regarded as two important physiological processes occurring upon cellular stress, which cooperate with each other to specify the cell fate‐survival or death in a mutually coordinated/exclusive manner, mediated by common initiators, effector modules and signaling pathways.^46^ Autophagy, originally thought to be a self‐destructive process through which the supernumerary or damaged cytoplasmic cargoes are delivered into lysosome for degradation, may counteract apoptosis to serve a prosurvival function.^47^ Actually, the close interplay between autophagy and apoptosis appears to be sophisticated in the sense that, autophagy constitutes an adaptive mechanism by which cells avoid the lethal event (as aforesaid, refraining apoptosis), yet in certain scenarios, it serves as an alternative approach to cellular demise (i.e., apoptotic promotion).^48^ In this regard, there are instances where autophagy monitors apoptosis, as a guardian or executioner depending in part on the tumor microenvironment, therapeutic intervention and the clinical stage of carcinoma. Notably, the role of autophagy in apoptosis is controversial and sometimes contradictory as both stimulatory and inhibitory effects have been found in NSCLC.^49^ For instance, disulfiram (DSF), a widely used alcohol deterrents, displays anticancer activity by promoting apoptosis in NSCLC cells, but this effect might be impaired by DSF‐driven accompanied protective autophagy.^50^ In contrast, Jervine (a sterodial alkaloid) inhibits NSCLC progression by inducing autophagy‐regulated apoptosis, and blocking autophagy abrogates this inhibitory effect on cell growth and apoptosis induction.^51^ With this research, we demonstrate that sertaconazole inhibits NSCLC cell growth by promoting complete autophagic flux, inhibition of which represses apoptosis and growth suppression induced by sertaconazole treatment, suggesting the proapoptotic and cytotoxic effect of sertaconazole‐induced autophagy in NSCLC cells.

Death receptors on cell surface, such as TNFR1 and Fas, function in apoptosis induction triggered by extrinsic stimuli.^52^, ^53^ TRADD acts as an adaptor protein to signal downstream of the TNFR1, which contains death domain required for the induction of apoptosis.^54^ After TNF stimulation, a transient intracellular complex (complex I) forms at TNFR1 to control the activation of RIPK1 for facilitating the subsequent apoptosis induction, and in this instance TRADD is the first protein to be recruited to complex I, suggesting its pivotal role in regulating apoptosis.^55^, ^56^ Recent study has also linked TRADD to autophagy, identifying TRADD as an important regulator of both autophagy and apoptosis, inhibition of which reduces apoptosis and thus restores cellular homeostasis by activating autophagy.^43^ In our study, we found that sertaconazole‐induced upregulation of TRADD triggers a complete autophagic flux to suppress NSCLC cell growth. Though this observation is apparently inconsistent with the aforementioned findings that TRADD induces apoptosis while inhibits autophagy, this paradox could largely be attributed to the different research models used, implying the context‐dependent function of TRADD in autophagy regulation which needs to be further investigated.

The Akt/mTOR pathway is one of the well‐recognized regulators of autophagy, as well as apoptosis.^57^, ^58^ Activation of this pathway functions in sustaining cancer cell survival not only via inhibition of apoptosis‐related genes and promotion of antiapoptotic proteins, but also by inhibiting autophagy to promote cell proliferation, especially under stress conditions, such as nutrient starvation or chemotherapy. Akt/mTOR promotes cell survival mainly through sequestering FoxOs away from the promoters of apoptotic genes.^59^ In result, the transcription of several proapoptotic genes such as BIM, Fas ligand and TRADD was reduced,^60^ indicating the negative regulatory role of Akt for TRADD expression. Intriguingly, it has been reported that anticancer drugs exhibit their killing effects partially by downregulating Akt signaling and enhancing the following TRADD expression.^41^ In the present study, we demonstrate that sertaconazole‐induced upregulation of TRADD decreases the phosphorylation of Akt, thereby promoting complete autophagic flux to further suppress the growth of NSCLC cells. It seems that the upstream and downstream relationship between TRADD and Akt is opposite when compared with the previous notions critically discussed. Regarding to this contradictory observation, we speculate that TRADD may display feedback regulation on Akt phosphorylation employing an uncharted mechanism yet to be further investigated.

## 4 CONCLUSION

Taken together, our results demonstrate that antifungal sertaconazole might be an efficacious antitumor drug for NSCLC treatment, and the induction of proapoptotic autophagy is identified as the key event for tumor inhibition in sertaconazole‐treated NSCLC cells. Of note, sertaconazole increases TRADD expression via protecting it from ubiquitination‐mediated degradation, thereby decreasing the phosphorylation of Akt to induce proapoptotic autophagy in NSCLC cells. These findings shed new light on the molecular event of sertaconazole‐induced NSCLC suppression by triggering TRADD‐regulated proapoptotic autophagy, which establishes a rational and promising strategy for NSCLC treatment by repurposing the antifungal sertaconazole.

## 5 METHODS

### 5.1 Cell culture

Human NSCLC cell lines (A549, H1299, H1975, HCC78, HCC827, H460, and PC‐9) and HEK293T, human bronchial epithelial cell line 16HBE were purchased from the American Type Culture Collection (ATCC). These cell lines were maintained in RPMI‐1640 or high glucose DMEM (Gibco) supplemented with 100 U/ml penicillin (MilliporeSigma), 100 U/ml streptomycin (MilliporeSigma), and 10% FBS (Biowest) in a humidified chamber at 37°C under 5% (v/v) CO_2_ atmosphere. All cell lines used in this experiment were cultured for less than 2 months before reinitiating from authentic stocks and were routinely regularly inspected by microscopic morphological observation and mycoplasma contamination.

### 5.2 Reagents and antibodies

Sertaconazole nitrate (HY‐B0736A), 3‐methyladenine (HY‐19312), Ferrostatin‐1 (HY‐100579), Z‐VAD‐FMK (HY‐16658B), and Necrostatin‐1 (HY‐15760) were obtained from Med Chem Express. MG‐132 (S2619) and CHX (S7418) were purchased from Selleck. DMSO (D2650) and chloroquine (C6628) were purchased from MilliporeSigma. Sertaconazole nitrate, Ferrostatin‐1, Z‐VAD‐FMK, and Necrostatin‐1 were dissolved in DMSO. Chloroquine and 3‐methyladenine were dissolved in PBS.

Antibodies: Caspase 3 (380189) and Cleaved‐caspase 3 (380189) were purchased from ZEN BIO. PARP (9532), Cleaved‐PARP (9532), phosphorylated (p‐)Akt (Ser473) (4060), p‐mTOR (Ser2448) (2971), p‐p70S6K (Ser371) (9208), p‐4EBP1 (Ser65) (9451), Akt (4685), mTOR (2972), p70S6K (9202), 4EBP1 (9452), Beclin 1 (3738), ATG5 (12994S), ATG7 (8558S), Bcl‐2 (15071), and ubiquitin (3936) were purchased from CST. TRADD (sc‐46635), β‐actin (sc‐1616), Ki67 (sc‐23900), HRP‐conjugated antimouse secondary antibody (sc‐2005), and HRP‐conjugated antirabbit secondary antibody (sc‐2004) were purchased from Santa Cruz Biotechnology. LC3 (NB100‐2220) was obtained from Novus. For immunofluorescence, goat antimouse Alexa Fluor 594 (A21044) and goat antirabbit Alexa Fluor 488 (A27034) were obtained from Invitrogen.

### 5.3 Detection of cell growth and proliferation

The short‐term effects of sertaconazole on cell growth were evaluated by MTT assay. Briefly, cells were plated in 96‐well plates at 5000 cells peer well and treated for 24 or 48 h, and then incubated with 5 mg/ml MTT (MilliporeSigma, M2128) for 4 h and dissolved in DMSO. The absorbance was measured at 570 nm with a spectrophotometer.

For colony formation assay, NSCLC cells were plated in 24‐well plates (500 cells/well) and then subjected to the indicated concentrations of drugs. After 2 weeks, the colonies were fixed using 4% paraformaldehyde and stained with 0.1% crystal violet, then photographed using a Molecular Imager Gel Do XR+ System (BIO‐RAD). The clone numbers were counted using Image J software.

EdU labeling was performed in 96‐well plates (4000 cells/well) to measure cell proliferation using the EdU Cell Proliferation Assay Kit. After indicated treatments, 10 μM EdU was added to the cells. Then the cells were incubated for 24 h at 37°C and fixed with 4% paraformaldehyde. DAPI was then added for nuclear staining followed by imaging with a fluorescence microscope (Axio Observer 7, ZEISS).

### 5.4 LDH release assay

Lactate dehydrogenase (LDH) test kit was used to detect the cytotoxicity. Cells were placed in 96‐well plates (6000 cells/well). After different treatment, the supernatant was transferred to the new 96‐well plate for LDH analysis according to the supplier's instruction.

### 5.5 Flow cytometry

Annexin V‐FITC/PI Detection Kit was used to examine apoptotic cells following the manufacturer's protocol. In brief, cells were trypsinized and washed twice with PBS, and then stained with PI or Annexin‐V for apoptosis analysis. At least 1 × 10^4^ live cells were collected by a FACSCalibur flow cytometer. Data were analyzed by FlowJo software.

### 5.6 TUNEL assay

Cells were cultured on glass coverslips in 24‐well plates (5000 cells/well) followed by periplocin treatment for 24 h at indicated concentrations. Cells were then fixed in 4% paraformaldehyde. The TUNEL staining was conducted using DeadEndTM Fluorometric TUNEL system following the manufacturer's instructions to detect apoptotic cells. Cells were photographed using a fluorescence microscope (Axio Observer 7, ZEISS), and the percentage of the TUNEL‐positive cells was evaluated.

### 5.7 Immunofluorescence

Cells were placed at 4 × 10^4^ cells/well on glass coverslips in 24‐well plates followed by transfection or treatment as indicated. The cells were washed with PBS and fixed with 4% paraformaldehyde. After exposure to PBS containing 0.4% Triton X‐100 and 5% BSA for 1.5 h, the slides were stained with primary antibodies at 4°C overnight, and then stained with Alexa Flour secondary antibodies at room temperature for 1 h. DAPI was used to stain nuclei for 8 min at room temperature. Images were visualized using a Zeiss LSM 710 confocal microscope.

### 5.8 Immunoblotting and immunoprecipitation

Cells were seeded in 6‐well plates at 2×10^5^ cells/well. Upon treatment, cells were harvested into an EP tube and digested in RIPA buffer in the presence of 1% protease inhibitor cocktail. The concentration of protein lysates was quantified using a bicinchoninic acid protein assay kit. Proteins were separated by SDS‐PAGE and transferred to a PVDF membrane. After blocking in 5% skimmed milk for 90 min, the samples were incubated in suitable primary antibody at 4°C overnight and the secondary antibody at room temperature for 90 min. Immunoreactive bands were detected by chemiluminescence reagent, with β‐actin as the internal control.

For immunoprecipitation assay, IP lysis buffer was used to lyse cells. The lysates were then incubated with 1 μg of the primary antibodies at 4°C overnight, followed by 4 h incubation with protein A/G agarose beads. After centrifugation, washing for 5 times and boiling with loading buffer, the immunoprecipitated proteins were analyzed by immunoblotting with the indicated antibodies.

The intensity of immunoblot bands was calculated by the ImgeJ software.

### 5.9 Quantitative RT‐PCR analysis

Total RNA was obtained using TRIzol. The PrimeScript™ RT reagent Kit was used to reverse transcribe total RNA (1 μg). The mRNA levels of indicated genes were quantified using the Bio‐Rad iTaq Universal SYBR Green Supermix (Bio‐Rad,1725271) in a CFX96 Real Time System (Bio‐Rad Laboratories). The primer pairs of TRADD were: forward 5‘‐TTCTGCGGCTATTGCTGA‐3′, reverse 5‘‐TGAAACTGTAAGGGCTGG‐3′.

### 5.10 RNA interference

All siRNAs were purchased from GenePharma (Shanghai, China) and transfected using PEI for 48 h following the manufacturer's protocol. The siRNA sequences were as follows: *Atg5* siRNA 5′‐GCAACUCUGGAUGGGAUUG‐3′; *BECN1* siRNA 5′‐CAGUUUGGCACAAUCAAUATT‐3′; *TRADD* siRNA 5′‐GGAGGAUGCGCUGCGAAAUUU‐3′.

### 5.11 Plasmids

The human TRADD coding region with C‐terminal Flag tag was ligated into the pcDNA3.1(+) vector. The PCR primers for TRADD were as follows: forward primer: 5′‐CGGGATCCATGGCAGCTGGGCAAAAT‐3′; reverse primer: 5′‐CCCTCGAGCTAGGCCAGGCCGCCATT‐3′.

### 5.12 Cellular thermal shift assay

Cells cultured in 100‐mm dishes to 80% confluency were treated with or without sertaconazole (30 μM) for 12 h. Cells were harvested by trypsin, resuspended with PBS, and then divided into 6 aliquots, each of which was heated at 51°C, 53°C, 55°C, 57°C, 59°C, 61°C for 5 min. Soluble fractions were then extracted by three cycles of freeze‐thawing with liquid nitrogen, followed by centrifugation at 17,000 × *g* for 10 min. Finally, samples were analyzed by immunoblotting with anti‐TRADD antibody.

### 5.13 Tumor xenograft model

Six‐week‐old male nude mice (BALB/c, 18–20 g each) were obtained from HFK Bioscience Co., Ltd. For generating xenograft model, A549 cells (1 × 10^7^ cells/mouse) were suspended in PBS and subcutaneously injected into flanks of mice. When the tumor volumes reached 100 mm^3^, mice were randomly divided into two groups intraperitoneally receiving 0.1 ml of vehicle (10% ricinus oil, 5% DMSO, 10% ethanol, 75% physiologic saline) or sertaconazole (75 mg/kg/day), respectively. The tumor volumes were measured every 2 days and calculated as (length × width^2^)/2. Mice were euthanized after 18 days and tumor tissues were formalin fixed immediately.

### 5.14 Statistical analysis

Statistical analysis was performed using GraphPad Prism 6.0 software. Statistical differences were determined using 2‐tailed Student's *t*‐test or two‐way ANOVA. Data were shown as mean ± SD. The *p*‐value was described as follows: *p < 0.05, **p < 0.01, ***p < 0.001.

## CONFLICT OF INTEREST

All the authors declare no conflict of interest.

## ETHICS APPROVAL

All animal studies were approved by the Institutional Animal Care and Treatment Committee of Sichuan University.

## AUTHOR CONTRIBUTIONS

JL and ZS conceived and designed the experiments, contributed new reagents, and supervised all the research. WZ, LZ and SQ performed the experiments. ZZ and XZ analyzed the data. WZ and LZ write the original manuscript. ZH and JJ revised the manuscript. All authors have approved the final version of the manuscript.

## Supporting information

Supporting Information

## Data Availability

All data are available from the corresponding authors upon request.
