# Supplementary material for: Sertaconazole provokes proapoptotic autophagy via stabilizing TRADD in nonsmall cell lung cancer cells
Source: MedComm (2020). 2021 Dec 16;2(4):821–37. doi: 10.1002/mco2.102 (PMC8706745; doi:10.1002/mco2.102)
Supplement: Supplementary file 1 — Supporting Information [file MCO2-2-821-s001.docx]

**Supplementary files**

**Sertaconazole Provokes Pro-apoptotic Autophagy via Stabilizing TRADD in Non-small Cell Lung Cancer Cells**

Wenhui Zhang^1#^, Li Zhou^2#^, Siyuan Qin^2^, Jingwen Jiang^2^, Zhao Huang^2^, Zhe Zhang^2^, Xiyu Zhang^3^, Zheng Shi^4*^, Jie Lin^1*^

**
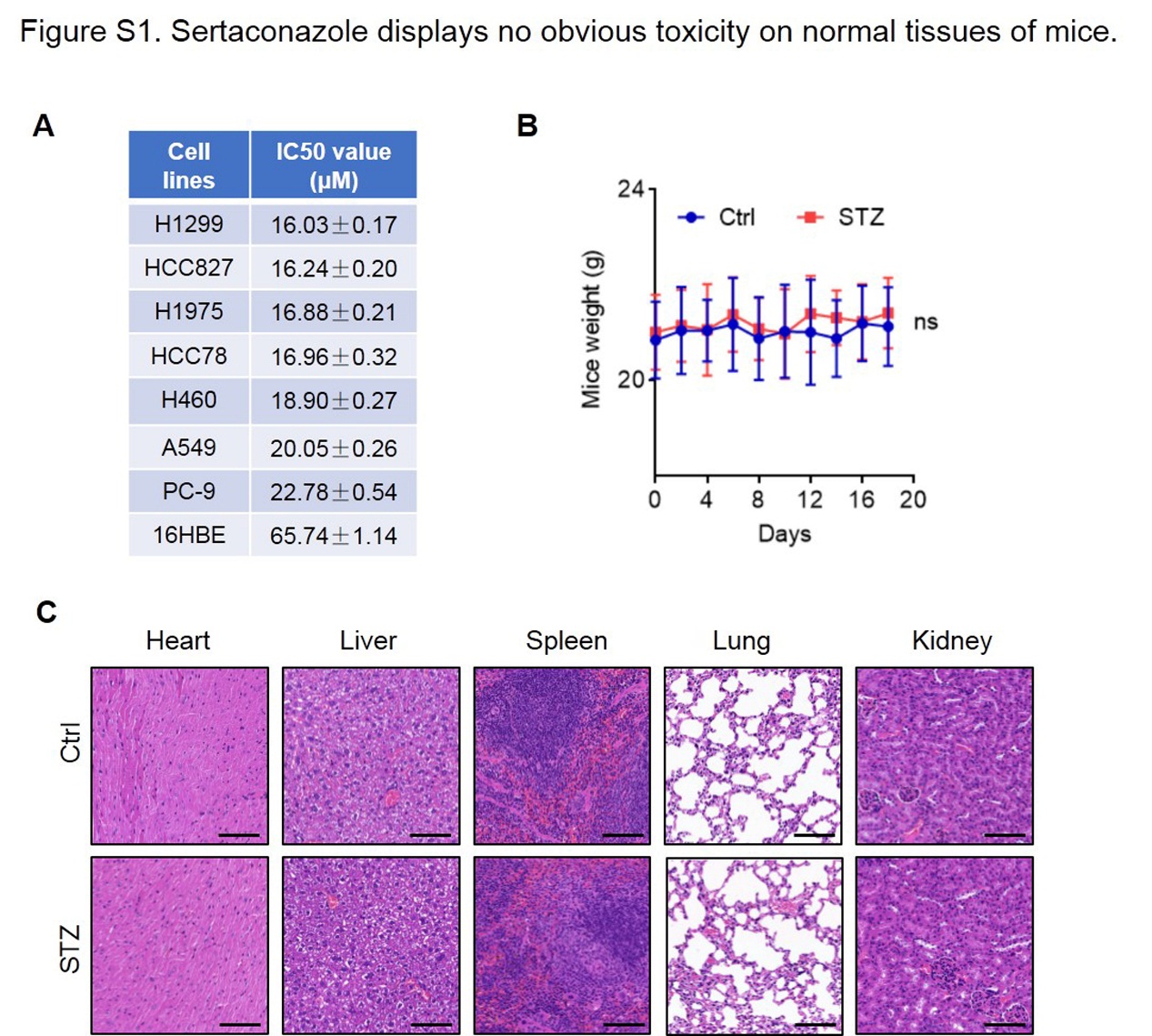
**

**Figure S1. Sertaconazole displays no obvious toxicity on normal tissues of mice.** **A**) IC50 values of sertaconazole in NSCLC cells and 16HBE cells. **B**) Body weight of tumor-bearing mice was monitored at the indicated time points. **C**) H&E staining of the heart, liver, spleen, lung, and kidney from mice. Scale bar, 50 μm. Statistic method: 2-tailed Student’s t-test. ns, not significant.

**
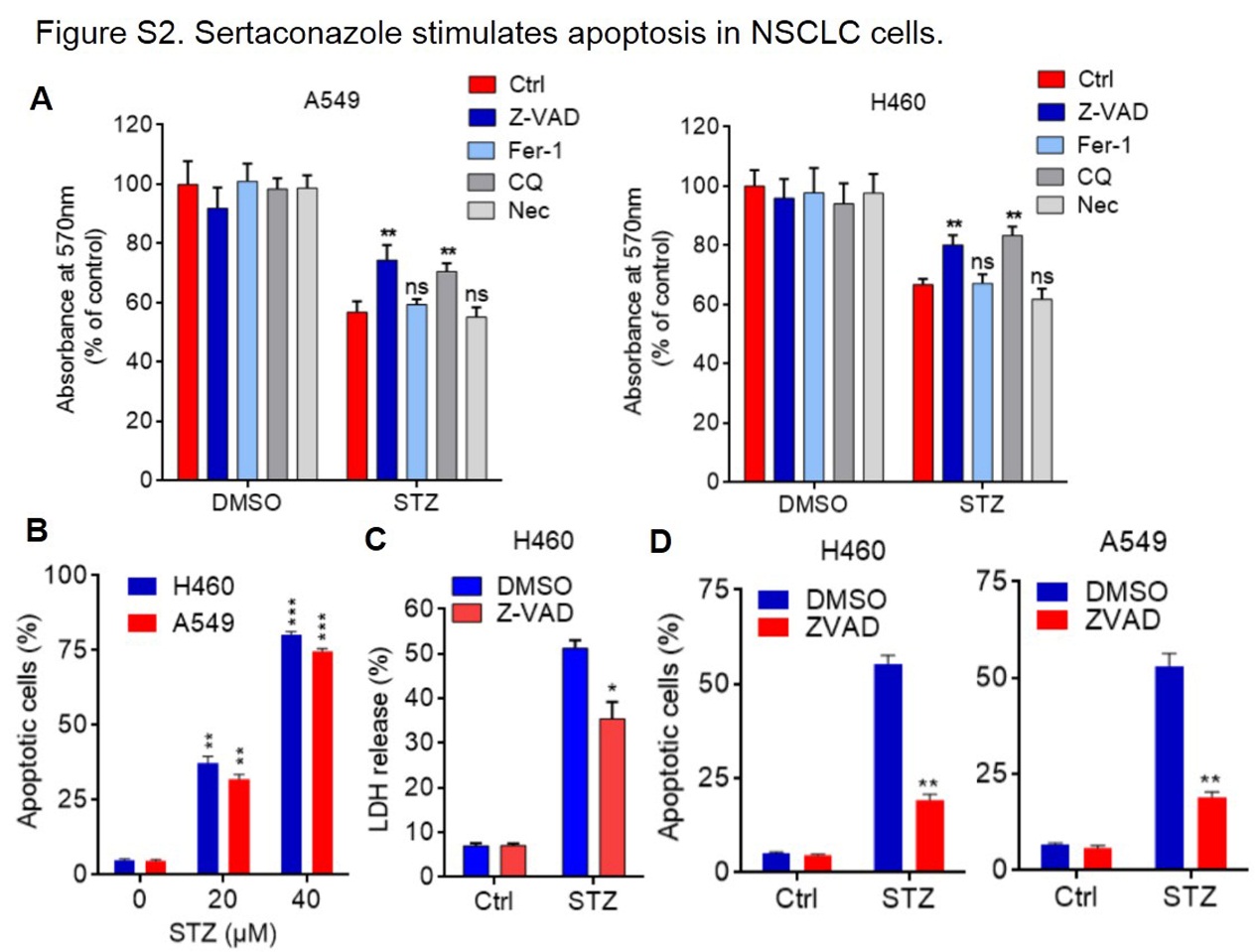
**

**Figure S2. Sertaconazole stimulates apoptosis in NSCLC cells**. **A**) MTT assay of A549 and H460 cells treated with the sertaconazole and in combination with or without Z-VAD, Fer-1, CQ, Nec for 24 h. Statistic method: Two-way ANOVA. **B**) Statistic analysis of apoptotic cells in (Figure 2A). Statistic method: 2-tailed Student’s t-test. **C**) H460 cells were treated with sertaconazole in combination with or without Z-VAD. LDH release assay was used to detect the cytotoxic effect of sertaconazole. Statistic method: Two-way ANOVA. **D**) Statistic analysis of apoptotic cells in (Figure 2H). Statistic method: Two-way ANOVA.

All experiments were repeated at least three times. Data are means with SD. ns, not significant, * *p*<0.05, ** *p*<0.01, *** *p*<0.001.


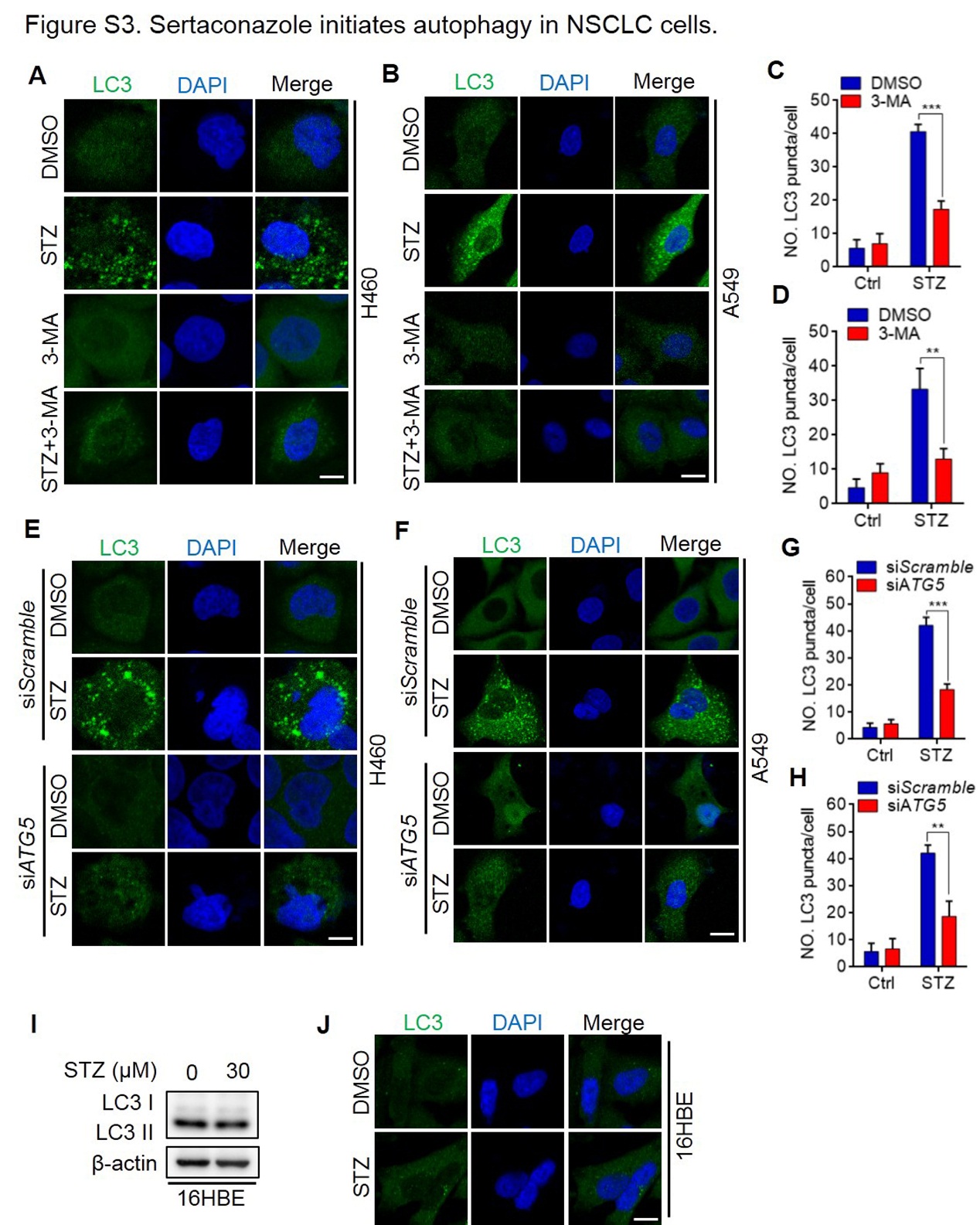


**Figure S3. Sertaconazole initiates autophagy in NSCLC cells.** **A-D**) A549 and H460 cells were treated with sertaconazole in the absence or presence of 3-MA for 24 h. Endogenous LC3 puncta were detected by immunofluorescent analysis. H460 (A, C), A549 (B, D). Scale bar, 10μm. Statistic method: Two-way ANOVA. **E-H**) Cells were transfected with si*Atg5* or si*Scramble*, followed by treatment with sertaconazole for 24 h. Endogenous LC3 puncta were detected by immunofluorescent analysis. H460 (E, G), A549 (F, H). Scale bar, 10μm. Statistic method: Two-way ANOVA. **I**) Immunoblotting of LC3 turnover in 16HBE cells treated with or without sertaconazole for 24 h. **J**) Immunofluorescence of endogenous LC3 puncta in 16HBE cells treated with or without sertaconazole for 24 h. Scale bar, 10 μm.

All experiments were repeated at least three times. Data are means with SD. ** *p*<0.01, *** *p*<0.001.


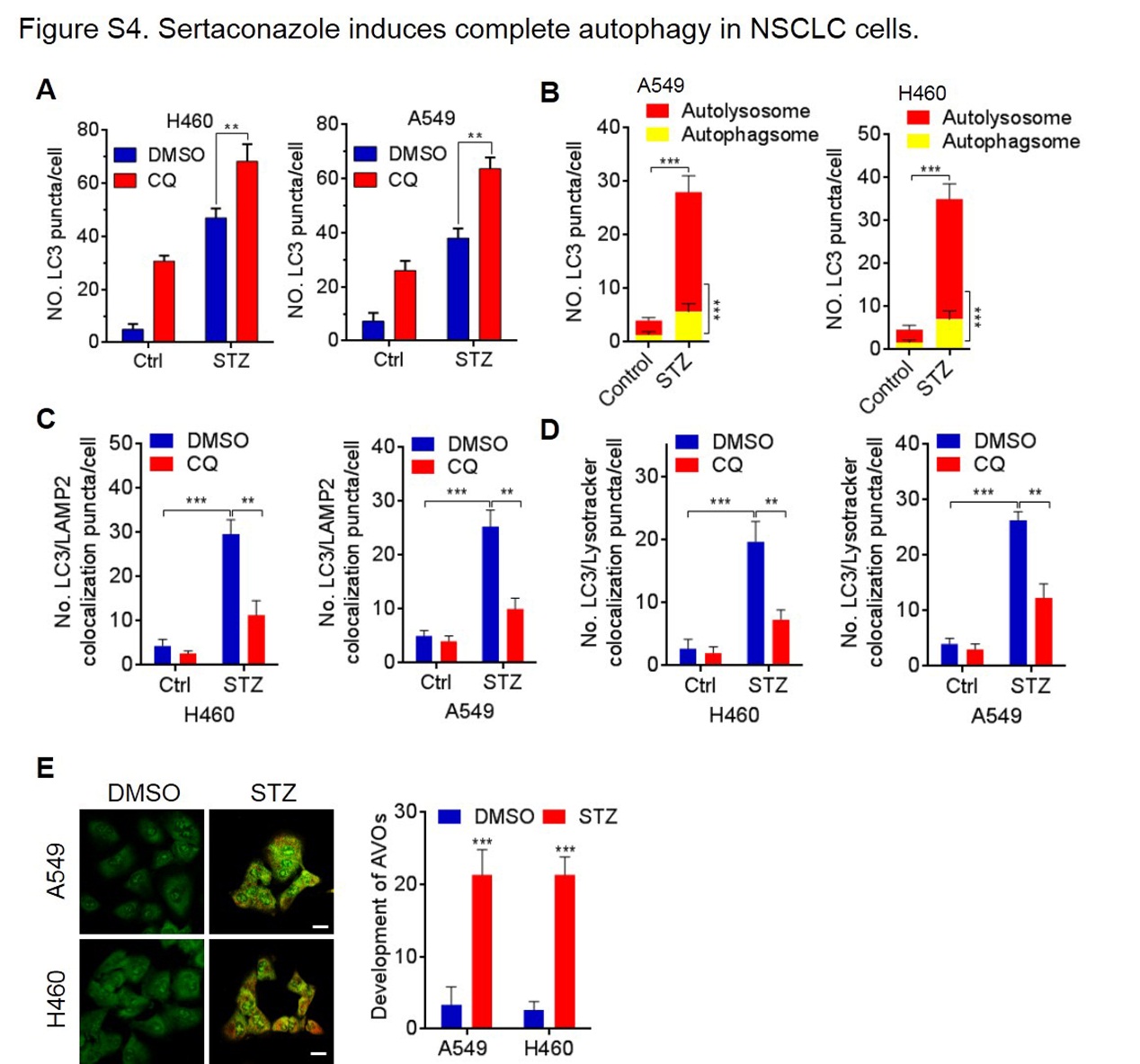


**Figure S4. Sertaconazole induces complete autophagy in NSCLC cells. A**) Statistic analysis of LC3 puncta in (Figure 4B). Statistic method: Two-way ANOVA. **B**) Statistic analysis of LC3 puncta in (Figure 4D). Statistic method: Two-way ANOVA. **C, D**) Statistic analysis of Figure 4E-H. Statistic method: Two-way ANOVA. **E**) Acridine orange staining of NSCLC cells treated with sertaconazole. Scale bar, 20μm. Statistic method: 2-tailed Student’s t-test.

All experiments were repeated at least three times. Data are means with SD. ** *p*<0.01, *** *p*<0.001.

**
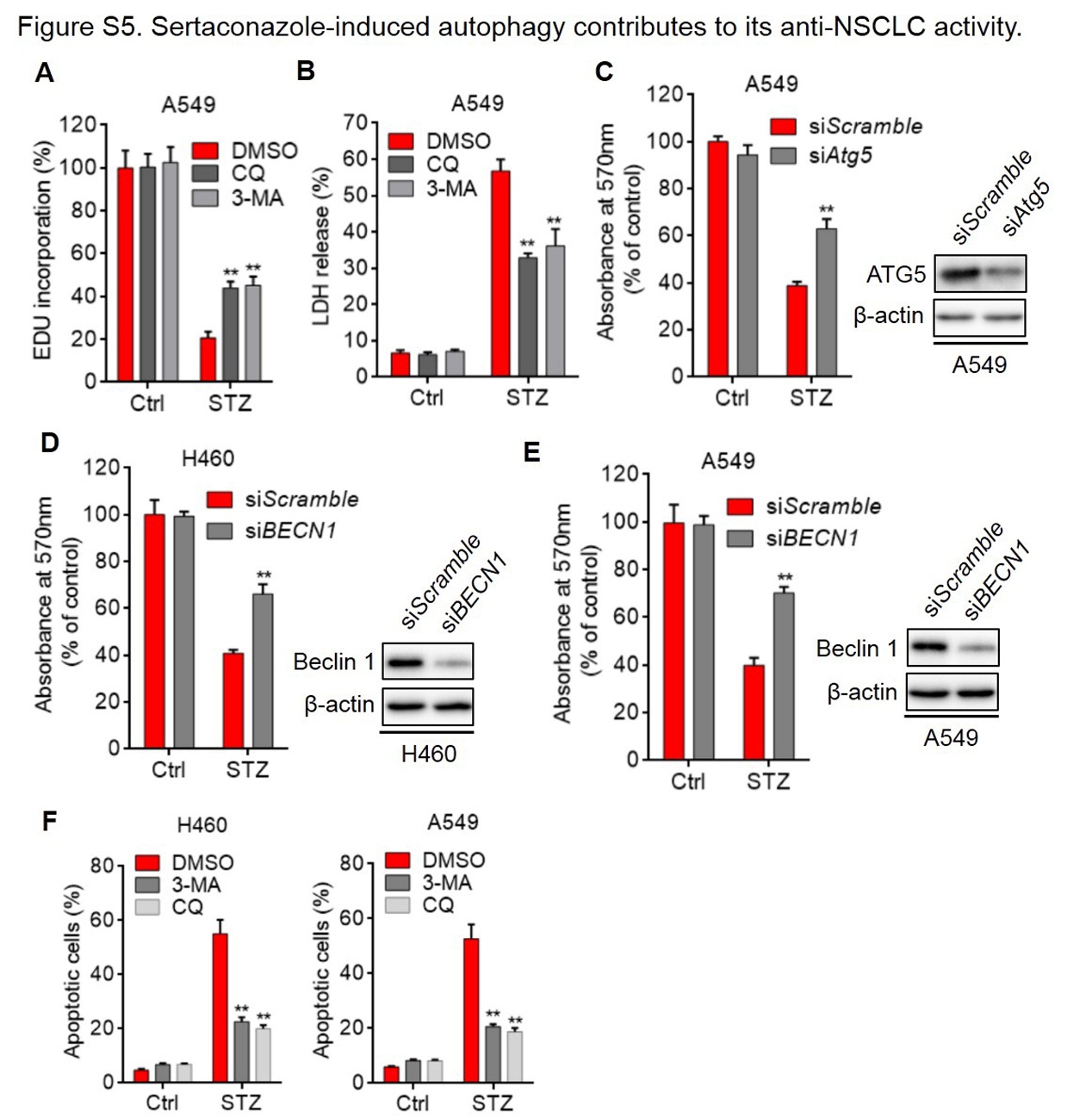
**

**Figure S5. Sertaconazole-induced autophagy contributes to its anti-NSCLC activity**. **A**) EdU incorporation assay was performed to detect the proliferation of A549 cells treated with sertaconazole in combination with or without 3-MA or CQ. Statistic method: Two-way ANOVA. **B**) A549 Cells were subjected to sertaconazole in combination with or without 3-MA or CQ, and cytotoxicity was detected by the release of LDH. Statistic method: Two-way ANOVA. **C**) A549 cells were transfected with si*Atg5* or si*Scramble*, followed by treatment with sertaconazole for 48 h. Cell growth was detected by MTT assay. Statistic method: Two-way ANOVA. **D, E**) H460 and A549 cells were transfected with si*Scramble* or si*BECN1*, followed by treatment with sertaconazole for 48 h. Cell growth was detected by MTT assay. Statistic method: Two-way ANOVA. **F**) Statistic analysis of apoptotic cells in Figure 5H. Statistic method: Two-way ANOVA.

All experiments were repeated at least three times. Data are means with SD. ** *p*<0.01.


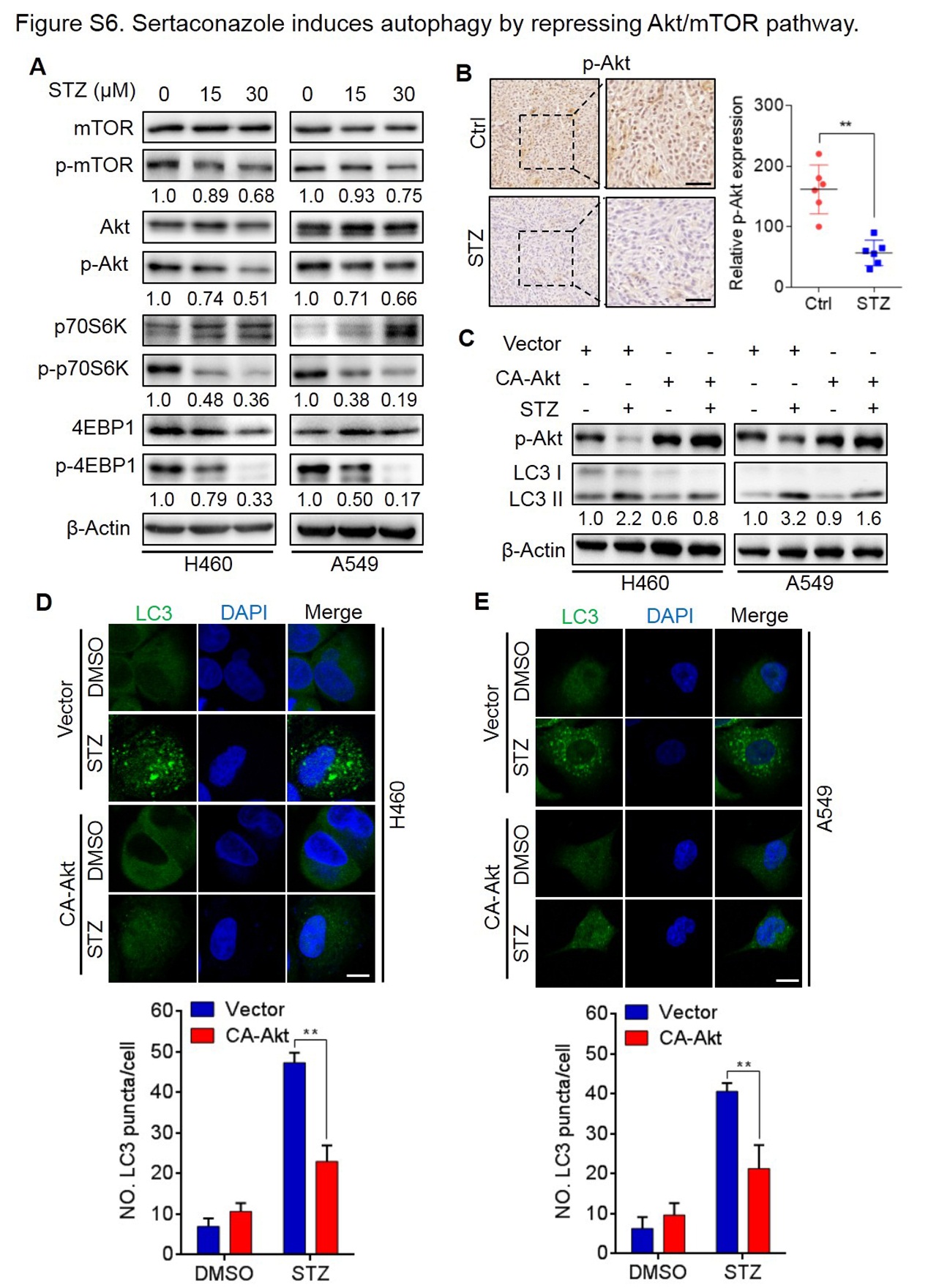


**Figure S6. Sertaconazole induces autophagy by repressing Akt/mTOR pathway. A**) Immunoblotting of phosphorylation of mTOR, Akt, 4EBP1 and p70S6K expression in NSCLC cells treated with sertaconazole for 24h. **B**) Immunohistochemical staining of p-Akt in tumor xenografts. Scale bar, 50 μm. Statistic method: 2-tailed Student’s t-test. **C-E**) H460 and A549 cells were transfected with or without CA-Akt plasmids, followed by treatment with sertaconazole for 24 h. Immunoblot analysis of LC3 turnover (C) and immunofluorescent analysis of endogenous LC3 puncta (D, E) were performed. Scale bar, 10 μm. Statistic method: Two-way ANOVA.

All experiments were repeated at least three times. Data are means with SD. ** *p*<0.01.


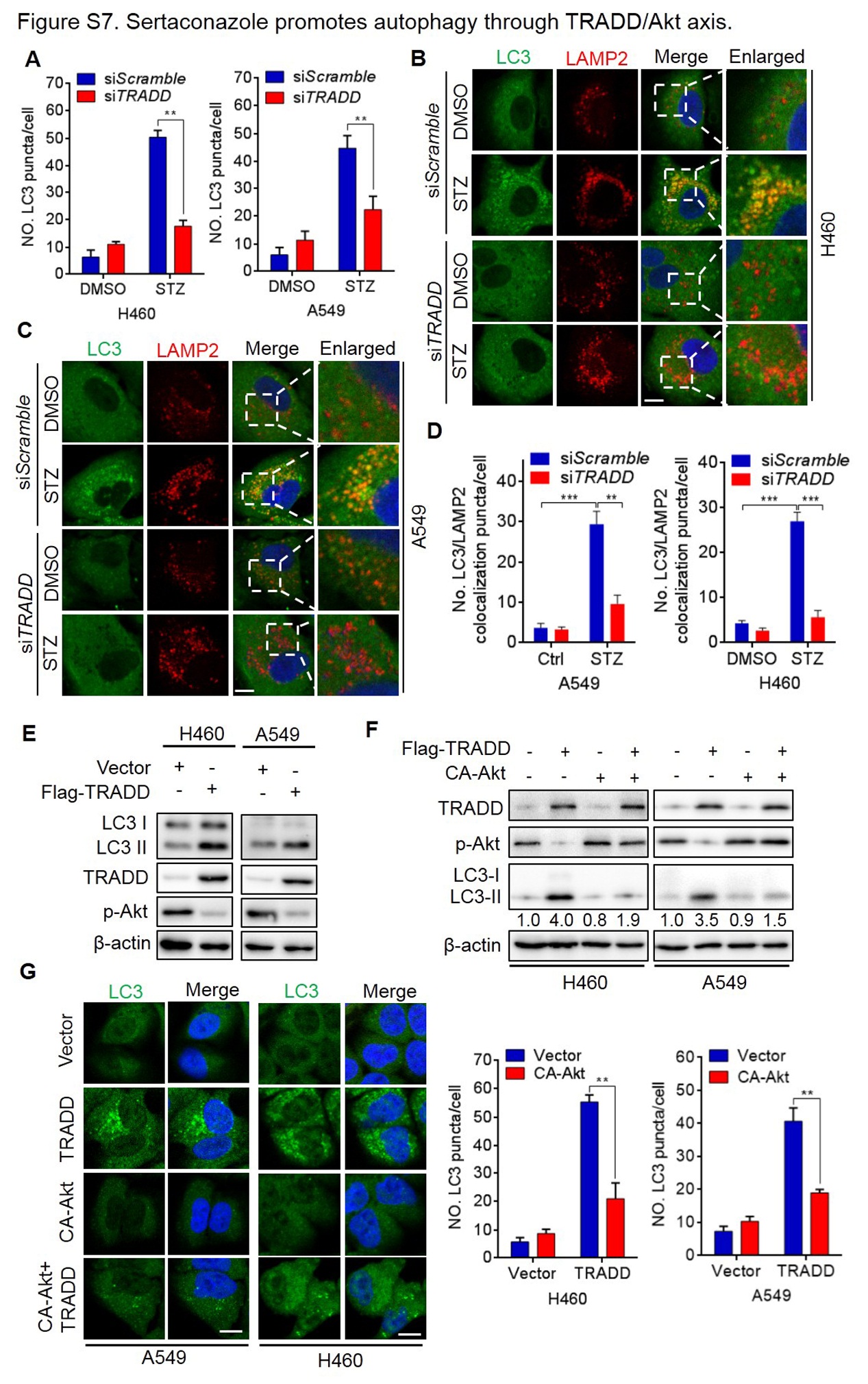


**Figure S7. Sertaconazole promotes autophagy through TRADD/Akt axis. A**) Quantification of LC3 puncta in (Figure 6I). Statistic method: Two-way ANOVA. **B-D**) H460 and A549 cells were transfected with si*TRADD* or si*Scramble*, followed by treatment with or without sertaconazole for 24 h. The colocalization of LC3 with LAMP2 was quantitated by immunofluorescent analysis. Scale bar, 10 μm. Statistic method: Two-way ANOVA. **E**) A549 and H460 cells were transfected with vector or Flag-TRADD plasmids. The protein expression of TRADD, p-Akt and LC3 was detected by immunoblot analysis. **F**) H460 and A549 cells were transfected with Flag-TRADD and CA-Akt plasmids, followed by treatment with sertaconazole for 24 h. The expression of TRADD, p-Akt and LC3 was detected by immunoblot analysis. **G**) Cells were treated as in (F). LC3 puncta were determined by immunofluorescent analysis. Scale bar, 10 μm. Statistic method: Two-way ANOVA.

All experiments were repeated at least three times. Data are means with SD. ** *p*<0.01, *** *p*<0.001.
